# A Record of Twenty-One Cases of Placenta Prævia

**Published:** 1883-12

**Authors:** Joseph Griffiths Swayne

**Affiliations:** Consulting Physician Accoucheur to the Bristol General Hospital; and Lecturer on Obstetric Medicine at the Bristol Medical School


### A RECORD OF TWENTY-ONE CASES OF PLACENTA PRÆVIA.

BY

JOSEPH GRIFFITHS SWAYNE, M.D.,

*Consulting Physician Accoucheur to the Bristol General Hospital; and Lecturer on Obstetric Medicine at the Bristol Medical School.*

---

PLACENTA PRÆVIA is a complication of labour which has always, with good reason, been dreaded by the accoucheur. There is no other complication which puts his skill and courage to so severe a test, and, except rupture of the uterus, there is none which is so dangerous. Fortunately it is rare. For this reason, as well as on account of its gravity, every case of placenta prævia is worth recording, and an experience of not more than a dozen cases becomes valuable. As I have met with more than a score since I commenced midwifery practice, and have made careful notes of each at the time of its occurrence, I am induced to present these notes in an abridged form in the accompanying table.

The rarity of placenta prævia is shown by the fact that in these twenty-one cases there was only one in which I was the original attendant: in all the others I was called in as consultant. Now, if I exclude all my consultation cases, I find that I have only met with one case of placenta prævia in a total of 1,437. This is even less than would appear from the statistics which have been given by Müller* as to the frequency of placenta prævia. By adding together the statistics of various in-

* Ludwig Müller *Placenta Prœvia,* Stuttgart, 1877.

### TWENTY-ONE CASES OF PLACENTA PRÆVIA.

| No. | Date. | Name and Residence. | Original Medical Attendant. | No of Pregnancy. | Presentation. | Complications. | Operative Treatment. | Result to Mother. | Result to Child. | Remarks. |
|---|---|---|---|---|---|---|---|---|---|---|
| 1 | 1851 | Mrs. S., Temple Street | Dr. Swayne ... ... | 21 | Partial placenta and head | Prolapse of cord | Rupture of membranes, replacement of cord | Recovered | Recovered | |
| 2 | 1853 | Mrs. L., Taylor's Ct., Union St. | Mr. Brock ... ... | Multi-para | Partial placenta and elbow | Prolapse of cord | Turning (by feet) ... | Recovered | Died ... | |
| 3 | 1855 | Mrs. —, Lewin's Mead | Midwife ... ... ... | Multi-para | Complete placenta | ... ... | Turning (by feet) ... | Recovered | Died ... | |
| 4 | 1857 | Mrs. S., Somerset Street | Mr. Green ... ... | 5 | Partial placenta and feet | Prolapse of cord | Rupture of membranes | Recovered | Died ... | Placenta expelled spontaneously before the child. |
| 5 | 1857 | Mrs. —, Jacob's Wells | Mr. McDonald ... | Multi-para | Partial placenta and head | ... ... | Rupture of membranes | Recovered | Recovered | |
| 6 | 1858 | Mrs. P., Lower Crescent | Mr. Sawer ... ... | 1 | Partial placenta and head | ... ... | Rupture of membranes, forceps | Recovered | Recovered | |
| 7 | 1860 | Mrs. P., Green Street | Mr. Baretti ... ... | 7 | Partial placenta and head | ... ... | Turning (by feet) ... | Recovered | Died ... | |
| 8 | 1862 | Mrs. —, Berkeley Place | Dr. Carter ... ... | Multi-para | Partial placenta, head and hand | ... ... | Cephalic version ... | Recovered | Died ... | 6½ months' child. |
| 9 | 1863 | Mrs. —, Lodge Street | Mr. Baretti ... ... | 1 | Partial placenta and head | Prolapse of cord | Turning (by feet) ... | Recovered | Died ... | |
| 10 | 1865 | Mrs. K., Hotwell Road | Mr. Crichton.. ... | 1 | Partial placenta and head | ... ... | Rupture of membranes | Recovered | Recovered | 8 months' child. |
| 11 | 1867 | Mrs. U., Horfield Road | Mr. Greenly ... ... | 6 | Partial placenta and head | ... ... | Turning (by feet) ... | Recovered | Died ... | Some pelvic deformity. |
| 12 | 1867 | Mrs. J., Leicester Place | Mr. Baretti ... ... | 3 | Complete placenta and head | Adherent placenta | Turning (by feet) ... | Recovered | Died ... | Had partial placenta prævia at second labour. |
| 13 | 1868 | Mrs. —, Broad Street | Mr. Willett ... ... | 9 | Complete placenta | ... ... | Turning (by feet) ... | Died ... | Died ... | She died during delivery from hæmorrhage and shock. |
| 14 | 1872 | Mrs. P., Clifton Wood | Mr. Baretti ... ... | 3 | Partial placenta and head | ... ... | Turning (by feet) ... | Recovered | Died ... | Some pelvic narrowing. |
| 15 | 1874 | Mrs. C., 21 Sion Hill | Mr. Baretti ... ... | 8 | Complete placenta & breech | ... ... | Bringing down feet ... | Died ... | Recovered | She died 7 days after labour from septicæmia. |
| 16 | 1874 | Mrs. L., 108 Pembroke Road | Mr. Coe ... ... ... | 5 | Partial placenta and knee | ... ... | Bringing down feet ... | Died ... | Died ... | She lived 2 months after delivery in very weak state, and at last died from pneumonia. |
| 17 | 1876 | Mrs. N., Beaumont Place | Mr. Willett ... ... | 9 | Partial placenta and head | ... .. | Rupture of membranes, forceps | Died ... | Died ... | She died 8 days after delivery from septicæmia. |
| 18 | 1876 | Mrs. B., Cornwallis Grove | Mr. S. H. Swayne | 10 | Partial placenta and head | Prolapse of cord | Turning (by feet) and craniotomy | Died ... | Died .. | She died of septicæmia 10 days after delivery. |
| 19 | 1876 | Mrs. M., Pill ... | Mr. Butter ... ... | 3 | Partial placenta and head | ... .. | Craniotomy ... ... | Died ... | Dead, putrid | She died 36 hours after delivery, apparently from shock. |
| 20 | 1877 | Mrs. F., Pembroke Road | Mr. Coe ... ... ... | Multi-para | Partial placenta and head | ... ... | Turning (by feet) ... | Died ... | Died ... | She lived 5 days after delivery and then died, apparently from embolism. |
| 21 | 1877 | Mrs. B., Long Ashton | Mr. Day... ... ... | 13 | Partial placenta Head, Feet | Twins, adherent placenta | Forceps 1st child, feet of 2nd child brought down | Died ... | Died ... Died | She died ¼ hour after delivery from shock. The adherent placenta was peeled off. |

vestigators he found reports of 813 instances in 876,432 births, or not quite one case in a thousand.

My own statistics also show a fact which had already been established by those of others, viz., that placenta prævia is much more frequent in multiparæ than in primiparæ. I have reported eighteen instances occurring in multiparæ and only three in primiparæ; this exactly corresponds with what has been observed by others, viz., that the proportion of the former to the latter class is six to one. Now I find that in all the cases I have attended of every kind the proportion of multiparæ is about 75 per cent., or as three to one, in comparison with the primiparæ. It has been observed that placenta prævia is most frequent in women who have borne children very rapidly, and in whom pregnancy has shortly followed abortion, both being conditions which favour relaxation of the uterine walls, dilatation of the cavity, and defective development of the decidua.

In the annexed table I have recognised two varieties of placenta prævia, viz., the partial and the complete. In the first, when the os uteri is sufficiently dilated, the finger feels on making an examination a portion of placenta and also the membranes covering the head or other presenting part; in the second nothing but placenta can be felt. I have recorded seventeen cases of the first variety to four of the second; thus showing that the partial placenta prævia is more than four times as frequent as the complete.

Another peculiarity which is shown in my table is the frequency with which abnormal presentations occur in conjunction with placenta prævia. Thus there were two presentations of the feet, one of the breech, one of the knee, one of the arm, and one of the head and hand; in addition to these there were no less than five cases in which the cord prolapsed. In Müller's statistics, in 1,148 cases there were 272 transverse and 107 breech presentations. The frequency of these anomalies is partly attributable to the large proportion of premature labours, and partly to the width and lax condition of the lower uterine segment and the consequent want of stability in the fœtus.

The *prognosis* as regards the mother is in cases of placenta prævia exceedingly unfavourable. There is a mortality of one mother in four during or shortly after delivery; including deaths from puerperal processes, Müller estimates the total mortality as not less than from thirty-six to forty per cent. My own statistics quite corroborate this conclusion, and show that Müller's estimate is by no means exaggerated if we follow up the history of the patient during the first two months after delivery. The dangers attending placenta prævia may be said to be "proximate" and "remote"; in the first there is the danger of death within a very few hours from hæmorrhage and shock, in the second within several days or weeks from septicæmia. In my own twenty-one cases there were eight maternal deaths; three of these died of shock within thirty-six hours, of these one died before delivery was completed, another a quarter of an hour after delivery, and another thirty-six hours after. Of the five who died at a later period from more remote causes, such as septicæmia, &c., one died at the end of five days from pulmonary embolism, three from septicæmia at the end of seven, eight, and ten days respectively, and one from pneumonia two months after delivery. It might appear to some to be over scrupulous to reckon this last case as a death from placenta prævia: but there is no doubt, I think, that the attack of pneumonia which ended fatally was indirectly due to that cause, as the woman remained in an exceedingly prostrate condition up to the time of the attack.

From my own figures it appears that the chief cause of death from placenta prævia is not hæmorrhage, but septicæmia; nor is this much to be wondered at. In the first place hæmorrhage predisposes to septicæmia by favouring absorption; in the next place, in these cases, the larger sinuses of the uterus are situated in the uterine neck, and this is a part that even in ordinary labour is liable to be forcibly stretched, bruised, and sometimes lacerated, and all this is still more likely to happen when turning is resorted to before the os is much dilated. Moreover the situation of the neck of the uterus renders it much more likely than the fundus to lie in contact with putrid clots and lochial discharge.

There is one peculiarity in my table that is worthy of notice, because it shows how untrustworthy statistical conclusions are which are derived from a small number of cases. It will be observed that in the first twelve cases all the mothers recovered; so that for sixteen years I went on attending such cases without losing a single patient, until at last I began to think that the danger of placenta prævia must be over-rated. But I was destined before long to be undeceived most completely. In the last nine cases only one mother recovered; but then these happened to be unusually bad cases, and five out of the eight deaths were from septicæmia and its consequences; remote causes of death which are very little under the control of the medical attendants.

The maternal mortality is also said to be twice as great in complete as in partial presentation of the placenta. My figures do not quite bear out this conclusion, but then this may arise from the limited number of my cases. I have met with four cases of complete presentation, with a maternal mortality of two, or just one-half; and seventeen partial with a maternal mortality of six, or rather more than one third.

As regards the infant, the prognosis is still more unfavourable than it is as regards the mother. According to Müller nearly two out of every three children are born dead, and more than one-half of those born alive die within the first ten days. In my own table only five children lived and seventeen died, two of these being twins; nor is this surprising when we consider *seriatim* the several risks which the child has to run. In all cases of placenta prævia there is more or less separation before birth of the organ which is the source of the intra-uterine life of the fœtus; and a more or less copious draining away of its life-blood. Then the child has often to incur the additional risk of turning, an operation which according to Dr. Churchill involves the loss of more than one child in three. Then, again, mal-presentations very frequently occur in cases of placenta prævia. I have recorded five cases of prolapsed cord; a mal-presentation the most dangerous of all to the children and involving a loss of more than one-half; I have also noted one transverse presentation and four of the inferior extremities. So it appears that according to Müller placenta prævia involves a loss of five children out of six, and according to my table of more than three out of four. I am led by this to the conclusion that there is no complication of labour which is so dangerous to the child.

As to the *treatment* of placenta prævia, it seems to be the almost unanimous opinion of the best obstetric authorities that the more complete the presentation of the placenta is the more necessity there is for prompt interference. On this account turning by the feet is almost invariably resorted to when there is a complete placental presentation. In my four cases of complete presentation turning was practised in three, but there was no necessity for it in the fourth, because the feet presented. Two mothers were saved and one child. Of the two mothers that died one lived a week and then died from septicæmia; whilst the other was moribund when I first saw her, turning having been half accomplished by the original attendant. In partial placenta prævia, when a very small portion presents, it is often sufficient merely to rupture the membranes and hasten delivery if necessary by the forceps. In this way six of my seventeen cases of partial presentation were treated, the result being that all the mothers recovered except one, who subsequently died from septicæmia, whilst four children were saved. It was necessary to apply the forceps in two of these cases. If, however, a larger portion of placenta presents, and the hæmorrhage is more urgent, more active interference, in the way of turning, becomes necessary. In this way eight of my cases of partial presentation were delivered; of these six mothers recovered and two died (from septicæmia after some days), whilst all the children were lost.

The balance of opinion, in the present day, seems to incline towards prompt interference as soon as a decided hæmorrhage has taken place, and to oppose an expectant plan in such cases, as formerly recommended; even although pregnancy may be some weeks short of the full term. My father had come to very much the same opinion as this, after more than forty years of extensive midwifery practice. It must always be remembered that there is no safety for the mother so long as pregnancy continues, and that, as Lusk remarks, "the fatality of placenta prævia is due not so much to the impotence of obstetric art as to the losses of blood which occur suddenly in the absence of professional assistance." On this account the induction of premature labour is strongly recommended after the thirty-second week of pregnancy, so soon as the diagnosis of placenta prævia is established, or at least with the occurrence of the first hæmorrhage. The best results have been obtained by Hecker, Hoffman, and Spiegelberg in accordance with this principle. I have had no cases of late years in which I could adopt this plan, but I certainly shall do so on the first opportunity. As Dr. Barnes remarks: "If the pregnancy have advanced beyond the seventh month, it will as a general rule, I think, be wise to proceed to delivery, for the next hæmorrhage may be fatal: we cannot tell the time or extent of its occurrence, and when it occurs, all, perhaps, that we shall have the opportunity of doing, will be to regret that we did not act when we had the chance."

